# HMGB1 cleavage by complement C1s and its potent anti-inflammatory product

**DOI:** 10.3389/fimmu.2023.1151731

**Published:** 2023-04-26

**Authors:** Marie Lorvellec, Anne Chouquet, Jonas Koch, Isabelle Bally, Luca Signor, Jeanne Vigne, Fabien Dalonneau, Nicole M. Thielens, Thierry Rabilloud, Bastien Dalzon, Véronique Rossi, Christine Gaboriaud

**Affiliations:** ^1^ Institute of Structural Biology (IBS), University Grenoble Alpes, CEA, CNRS, Grenoble, France; ^2^ Chemistry and Biology of Metals, University Grenoble Alpes, CNRS UMR 5249, CEA, IRIG-LCBM, Grenoble, France

**Keywords:** complement system, HMGB1 alarmin, cytokines, C1 protease, RAW264.7 cells, macrophage, inflammation, interleukin 6

## Abstract

Complement C1s association with the pathogenesis of several diseases cannot be simply explained only by considering its main role in activating the classical complement pathway. This suggests that non-canonical functions are to be deciphered for this protease. Here the focus is on C1s cleavage of HMGB1 as an auxiliary target. HMGB1 is a chromatin non-histone nuclear protein, which exerts in fact multiple functions depending on its location and its post-translational modifications. In the extracellular compartment, HMGB1 can amplify immune and inflammatory responses to danger associated molecular patterns, in health and disease. Among possible regulatory mechanisms, proteolytic processing could be highly relevant for HMGB1 functional modulation. The unique properties of HMGB1 cleavage by C1s are analyzed in details. For example, C1s cannot cleave the HMGB1 A-box fragment, which has been described in the literature as an inhibitor/antagonist of HMGB1. By mass spectrometry, C1s cleavage was experimentally identified to occur after lysine on position 65, 128 and 172 in HMGB1. Compared to previously identified C1s cleavage sites, the ones identified here are uncommon, and their analysis suggests that local conformational changes are required before cleavage at certain positions. This is in line with the observation that HMGB1 cleavage by C1s is far slower when compared to human neutrophil elastase. Recombinant expression of cleavage fragments and site-directed mutagenesis were used to confirm these results and to explore how the output of C1s cleavage on HMGB1 is finely modulated by the molecular environment. Furthermore, knowing the antagonist effect of the isolated recombinant A-box subdomain in several pathophysiological contexts, we wondered if C1s cleavage could generate natural antagonist fragments. As a functional readout, IL-6 secretion following moderate LPS activation of RAW264.7 macrophage was investigated, using LPS alone or in complex with HMGB1 or some recombinant fragments. This study revealed that a N-terminal fragment released by C1s cleavage bears stronger antagonist properties as compared to the A-box, which was not expected. We discuss how this fragment could provide a potent brake for the inflammatory process, opening the way to dampen inflammation.

## 1 Introduction

Discovered as a chromatin non-histone nuclear protein (1), HMGB1 exerts in fact multiple functions depending on its location and its post-translational modifications (2). Intracellularly, HMGB1 can shuttle between the nucleus, its main location, and cytoplasm, in response to stress signals (3). In the nucleus, HMGB1 binds and bends DNA to regulate chromatin structure and transcription. It is involved in DNA replication, repair, and recombination (4, 5). However, HMGB1 can also be actively secreted by immune cells during inflammation or passively released by late-apoptotic or necrotic cells (6). Whithin this abnormal extracellular location, HMGB1 is perceived by the immune system as an alarmin or damage associated molecular pattern (DAMP) (7). In this context, HMGB1 acts in synergy with endogenous and exogenous danger signals to promote inflammation (8). Indeed, HMGB1 acts as an enhancer, triggering inflammation through complex association with minute amounts of other DAMP molecules, although it does not possess proinflammatory activity on its own (9). HMGB1 was also reported to impair efferocytosis (10, 11) and to be involved in mediating immune tolerance of apoptotic cells or cancer cells through RAGE receptor (12, 13). Reversely, a set of DAMP molecules, including HMGB1, may target cancer cells to immunogenic cell death (14, 15). These and other HMGB1 functions impact immune and inflammatory responses in health and diseases, from signaling to resolution and repair steps (16–21).

As location is a key determinant of HMGB1 function, it is modulated by several intracellular modifications involved in the mechanisms of its secretion and release, which have been studied in depth (3). Its functional regulation has mainly been explored regarding the impact of the oxidation state of its three cysteines (8). Among other possible regulatory mechanisms, proteolytic processing could be highly relevant for HMGB1 catabolism and functional alteration in the context of inflammatory diseases (22). Since HMGB1 plays a critical role in the pathophysiology of many inflammatory disorders, this question about additional functional modulation needs further investigations (23). Knowing that the complement C1s protease can cleave the nuclear alarmin HMGB1 *in vitro*, it could modulate its functional activity *in vivo* (24, 25). Indeed, C1s cleavage was shown to impact the inflammatory action of HMGB1 on macrophages and dendritic cells, as well as to play a possible role in limiting the presence of nuclear autoantigens (25). However, the underlying molecular mechanisms remain undefined.

**Figure 1:**
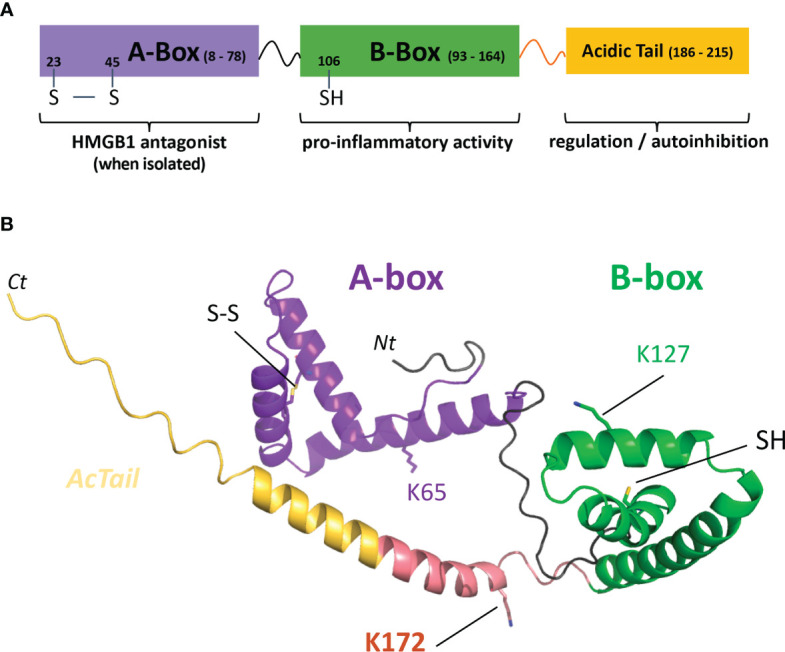
HMGB1 modular structure, C1s cleavage sites and predicted model. **(A)** The HMGB1 modular structure mainly includes two DNA-binding boxes, A-box (purple) and B-box (green), as well as a C-terminal negatively charged tail regulating its activity (yellow). The transition between the B-box and the acid stretch is shown in salmon pink. **(B)** The predicted model used was obtained from the AlphaFold Protein Structure database (26). However, it should be noted that the structure after the B-box (including the AcTail and the salmon pink transition segment) is highly uncertain and flexible, as well as the relative orientation between the two boxes. The salmon pink region includes the main C1s cleavage site after K172, as will be shown in this study. C1s also secondary cleaves HMGB1 after K65 and K127 (lysine side chains shown in stick). The disulfide bond between C23 and C45 in the A-box (SS) and the B-box C106 (SH) are also shown in stick. UNIPROT residue numbering is used throughout the text. *Nt, Ct*: chain extremities.

Structurally, HMGB1 is a 215 amino acid long protein subdivided into three main functional parts, which are the A-box and B-box, two DNA-binding domains, and a C-terminal acidic-tail (AcTail), which is enriched in glutamate and aspartate residues repeats (**Figure 1**). According to previous studies using truncated constructs of HMGB1, the B-box harbors the ability to induce cytokine secretion while the A-box alone shows antagonist effects with full length HMGB1 (9, 27, 28). As previously mentioned above, the oxidation state of its three cysteines regulates the biological activity of HMGB1 in the extracellular environment (8). Two cysteines in the A-box (C23, C45) can form a disulfide bond and the third cysteine (C106) is in the B-box (**Figure 1**).

The complement C1s protease was initially discovered as part of the host front line immune response. Its best characterized function is to activate the complement system through the classical pathway (CP), as part of the complement C1 complex. In this complex, C1s is associated to the C1r protease, and the C1r2C1s2 tetramer associates with the recognition protein C1q. Upon C1 activation on a target surface, C1s gets activated by C1r, and then triggers the CP activation by cleaving complement C4 and C2, under the control of C1-inhibitor (29–31). CP is involved in the control of bacterial infections, for example by *S. pneumoniae* (32, 33), and it plays also an essential role in the clearance of immune complexes and apoptotic cells (34). However, complete genetic deficiencies of CP components lead to a strong susceptibility to develop the systemic lupus erythematosus (SLE) autoimmune disease (35). This led to the hypothesis that the CP proteins protect against SLE, suggesting a major role in immune tolerance for these proteins, which has motivated further studies to decipher their possible functional implications, especially for C1q (36). However, this question still needs to be addressed for C1s. Indeed, rare complete genetic C1s deficiencies also lead to high susceptibility to develop SLE, in addition to higher susceptibility to infections, with high morbidity at a young age (37). Furthermore, C1r and C1s heterozygous mutations in periodontal Ehlers-Danlos syndrome (pEDS) suggest a gain of function of C1s which could be deleterious in collagen tissue homeostasis as well as in periodontal tissue maintenance (38). HMGB1 cleavage by C1s could be among the C1s accessory enzymatic activities possibly supporting a protective role regarding SLE or a destructive role in pEDS.

This study details where HMGB1 gets cleaved by C1s, at the residue level, and why this result is unexpected. It also shows examples on how the molecular environment modulates the output of this enzymatic reaction. Moreover, it reveals quite unexpectedly the potent anti-inflammatory potential of F3, a major A-box fragment released by C1s cleavage. These results will be discussed in terms of enzymatic modulation of HMGB1 function and how this might open therapeutic perspectives.

## 2 Material and methods

### 2.1 Proteins, cells and reagents

The C1 proteins C1s and C1r have been purified from human plasma or recombinantly according to published procedures (24, 29). Recombinant C1s (rC1s) was activated by active serum C1r before use, and the activation rate checked by SDS-PAGE analysis under reducing conditions (24). Human neutrophil elastase (HNE) was obtained from Elastin Products Company, Inc. HiTrap affinity and Hiload Superdex columns were from GE Healthcare Life sciences, (Velizy-Villacoublay, France). TBS solution was either obtained from Euromedex or prepared as follows: 25 mM Tris, 150 mM NaCl, pH 7.5.

Site-directed mutagenesis was performed using the QuickChange II XL kit (Agilent Technologies, Les Ullis, France). The sequence integrity of all constructs and mutants was confirmed by DNA sequencing analyses (Genewiz/Azenta, Leipzig, Germany).

### 2.2 Construction, production and purification of recombinant HMGB1 and its variants, and of recombinant complement MASP-2

#### 2.2.1 HMGB1 WT and variants

The modular structural features of HMGB1 WT are described in **Figure 1**. The initial plasmid coding for HMGB1 WT has been optimized for bacterial production and contains a C-terminal His (7) tag added for purification purpose (**Figure S1**). HMGB1 variants have been engineered, and their sequences are given in **Figure S1**. The plasmids coding for the A-box and its derivative rF3(1-K65) have a N-terminal His (7) tag and are not optimized for bacterial production. As previously described (24), the recombinant production of HMGB1 has been performed in *E. coli* BL21(DE3) cells (Sigma-Aldrich), transformed with a pET-28a plasmid coding for HMGB1 or its variants. The cells were grown in an autoinduction medium (39), except in the case of the 3S variant, for which the conventional IPTG induction has been used. After lysis of the cells by sonication, AKTA Fast Protein Liquid Chromatography (FPLC) setup was used for protein purification in three steps. The first step is usually a tag affinity separation using a hiTRAP Chelating HP 5 ml column. A second step on a hiTRAP heparin HP 5 ml column is used to remove excess DNA bound to the HMGB1 boxes. The final step is a gel filtration using a Hiload Superdex 75 16/600 column. Because of different biochemical properties of the HMGB1 rF2(1-K128) variant, the first two steps had to be reversed to purify this fragment, and affinity on a Q-sepharose column was used for the last purification step. The parameters used to measure the concentration of HMGB1 and its variants are given in **Table S1**.

#### 2.2.2 Recombinant MASP-2

Recombinant human MASP-2 was produced in HEK293-F cells as described for human C1s (24) using a pcDNA3.1-MASP-2-Flag expression vector. Briefly, a DNA fragment encoding the MASP-2 signal peptide, the mature protein, and an additional C-terminal Arg-(His)6 sequence (MASP-2-RH_6_) was excised from the pFast-Bac-MASP-2-RH_6_ plasmid (40) and inserted between the BamHI and EcoRI sites of the pcDNA3.1/Neo(+) plasmid (Thermofisher Scientific). Replacement of the RH_6_ tag by a Flag tag (DYKDDDDK) was performed by site-directed mutagenesis (Quick Change XL site-directed mutagenesis kit, Agilent Technologies). The pcDNA3.1-MASP-2-Flag construct was used for stable transfection of HEK293-F cells grown in FreeStyle 293 medium using 400 μg/ml neomycin (Thermofisher Scientific). Recombinant MASP-2 was purified from 500 ml cell culture supernatant on a 2 ml anti-FLAG M1 agarose column (Sigma-Aldrich) as described by Bally et al. (41). Most of the protein was recovered in the flow through and further purified by affinity chromatography on a C1q-Sepharose column, as described for purification of recombinant MASP-2 expressed in S2 Drosophila cells (42), except that 25 mM Tris was used instead of 50 mM triethanolamine in the buffers. The parameters used to measure MASP-2 concentration are given in **Table S2**.

### 2.3 Enzymatic digestion of HMGB1 and its variants

HMGB1, its variants or fragments were submitted to C1s digestion using various enzyme/substrate (E/S) molar ratio in 50 mM Tris, 150 mM NaCl, pH 7.4, for 100 min at 37°C. The reaction products were then analyzed by SDS-PAGE in reducing conditions (see section 2.4.1). Various HMGB1 amounts, E/S ratios, incubation times were used to match appropriate conditions for the different steps of the analysis. For example, the molar E/S ratio was between 5 and 20%, 2 to 6-7 μg HMGB1 were digested by C1s, and the incubation time varied from 90 min (simple SDS gel analysis) to 5-7 h for the MS analyses that required more complete digestion.

**Figure 2:**
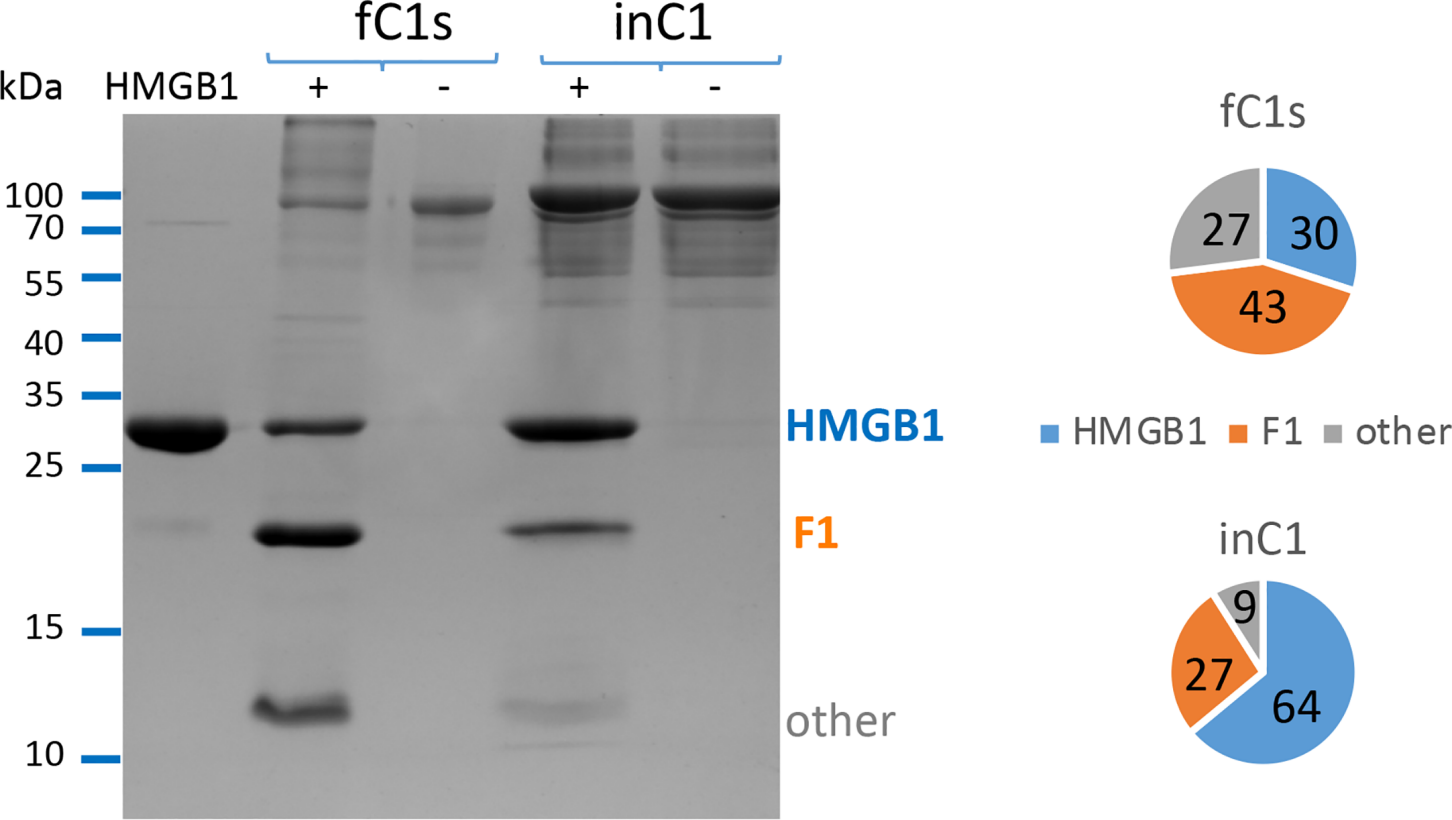
Free C1s (fC1s) cleaves HMGB1 more efficiently than C1s inside the C1 complex (in C1). The different proportions of remaining HMGB1 (blue), fragment F1 (orange) and other cleavage products (grey) are illustrated using a representative SDS-PAGE analysis performed at 10% E/S ratio (23 pmoles of C1s for 6 μg HMGB1). A summary view of the common trend is given using the ratios computed from the mean band intensity scanned in 3 independent measures performed at 5 or 10% E/S ratio, during 100 min at 37°C. In order to prevent C1 complex dissociation upon dilution, C1 concentration was set at 0.25 or 0.5 μM (respectively for the 5 or 10% molar ratio) to perform these experiments, with 1mM calcium chloride.

In order to compare the C1s enzymatic activity, free or within the C1 complex, we aimed to use the same C1s molar concentration and secure C1 assembly using C1 concentrations above 0.25 μM. Serum C1q (0.25 μM), C1r (0.5 μM) and recombinant activated C1s (0.5 μM) were mixed to form the C1 complex (0.25 μM), and incubated 90 min at 37°C for its activation. Comparative cleavage experiments (100 min, 37°C, same conditions as above) were then performed on 6 μg HMGB1 with 10% C1s either free or from C1 prepared at 0.25 μM (**Figure 2**). The same quantities of activated C1s (e.g. 23 pmoles for 10% E/S) were used in all conditions. The same analysis was also performed twice using 5% C1s (free or within C1).

For the enzyme comparison, HMGB1 was mixed with C1s or Human Neutrophil Elastase (HNE) at a 2.5% E/S molar ratio in TBS and incubated at 37°C for 90 min (SDS-PAGE analysis) or 5 h at 10% (SDS-PAGE and MS analyses). Further enzymatic comparison was performed using the C1r and MASP-2 proteases at a 10% E/S molar ratio to digest HMGB1 WT at 37°C for 90 min. For the SDS-PAGE analysis, reactions were stopped by addition of Tricine SDS Sample Buffer and reduced with *β*-mercapto-ethanol (0.35 M) and heated at 95°C for 10 min (or 85°C for 5 min for Tricine gels). The digestion mix was analysed by SDS-PAGE, further described in section 2.4.1.

### 2.4 Biochemical characterization

Protein concentrations were estimated using the parameters (Mw and A_1%, 1 cm_ at 280 nm) described in **Tables S1**, **S2**.

#### 2.4.1 SDS-PAGE and western blot analyses

HMGB1 and its cleavage products were initially analyzed by SDS-PAGE under reducing conditions using 14% poly-acrylamide Tris/HCl gels and then colored using Instant-Blue (#119211, Expedeon, Abcam, Paris, France). The use of Tricine SDS gels 10-20% (#12090106, Invitrogen) was implemented for this study to better distinguish HMGB1 fragments between 7 and 20 kDa.

For the western blot (WB) analyses, 7 pmol of HMGB1 (full-length or fragments) was incubated with C1s (see Enzymatic Reactions) and separated on Tricine gels. Proteins were transferred on nitrocellulose membranes using the semi-wet Trans-Blot Turbo transfer system (BioRad). Membranes were blocked with TBS 5% milk (w/v). The blocked membranes were probed with primary antibodies (rabbit anti-HMGB1-Nter, dilution 1:1000, #H9664, Sigma-Aldrich) and anti-HIS coupled to peroxidase (dilution 1:3000, #A7058, Sigma-Aldrich) for 2 h at room temperature in TBS (Euromedex) 5% milk (w/v). Membranes were washed 3 times with TBS 0.05% Tween (w/v). The anti-HMGB1-Nter probed membrane was incubated with the secondary antibody (goat anti-rabbit IgG coupled to peroxidase, dilution 1:20000, #A0545 Sigma-Aldrich). Membranes were revealed with an enhanced chemiluminescence detection kit (GE healthcare), scanned using a ChemiDoc apparatus (BioRad) and analyzed with ImageLab (BioRad).

#### 2.4.2 LC/ESI mass spectrometry

Liquid Chromatography Electrospray Ionization Mass Spectrometry (LC/ESI-MS) was performed on a 6210 LC-TOF spectrometer coupled to a HPLC system (Agilent Technologies). All solvents used were HPLC grade (Chromasolv, Sigma-Aldrich), trifluoroacetic acid (TFA) was from Acros Organics (puriss., p.a.). Just before analysis protein samples were diluted in acidic denaturing conditions to a final concentration of 5 µM with solution A (0.03% TFA in water). Solvent B was 95% acetonitrile-5% water-0.03% TFA. Protein samples were firstly desalted on a reverse phase-C8 cartridge (Zorbax 300SB-C8, 5 μm, 300 µm ID´5 mm, Agilent Technologies) for 3 min at a flow rate of 50 μl/min with 100% solvent A and then eluted and separated onto a RP-C8 column (Jupiter, 5 μm, 300 Å, 1 mm ID × 50 mm, Phenomenex) at a flow rate of 50 μl/min using the following linear gradient: from 5 to 95% solvent B in 15 min, then remaining 2 min at 100% solvent B and finally re-equilibrating the column at 5% solvent B for 10 min. MS acquisition was carried out in the positive ion mode in the 300-3200 *m/z* range. MS spectra were acquired and the data processed with MassHunter workstation software (v. B.02.00, Agilent Technologies) and with GPMAW software (v. 7.00b2, Lighthouse Data, Denmark).

### 2.5 RAW264.7 macrophage cells stimulation

#### 2.5.1 Cell culture

RAW264.7 were sub-cultured every two days using RPMI medium supplemented with 10% of decomplemented FBS (heated at 56°C for 40 min), 10 mM of Hepes solution (#H0887, Sigma-Aldrich) and 5 µg/ml ciprofloxacin (#17850, Sigma-Aldrich).

#### 2.5.2 Stimulation protocol

Before stimulation, cells were seeded at 300 000 cells/ml in 6-well adherent plates (#353046, Falcon) with 2 ml/well and incubated for 2 days at 37°C, 5% CO_2._ For the stimulation, LPS (#L2880, Sigma-Aldrich) was used alone or in complex with HMGB1 WT, A-box or rF3. HMGB1 and its variants were produced and stored at -20°C in TBS solution, whereas LPS was stored in H_2_O + 30% ethanol. 24 h prior to stimulation, HMGB1 and LPS were mixed to form complexes at 4°C. The complexes were then diluted in medium to stimulate cells at a final concentration of 0.47 pmol/ml, 0.94 pmol/ml or 1.88 pmol/ml for HMGB1 and 1 ng/ml or 2 ng/ml of LPS. Cells were stimulated for 24 h at 37°C, 5% CO_2_. Cells supernatants were harvested and stored at -20°C for later analysis.

#### 2.5.3 Cell physiological state assessment

The levels of cell viability and phagocytosis were analyzed (data not shown) by flow cytometry (FACSCalibur™ – BD Bioscience). The phagocytosis level was assessed by 3 h incubation with fluorescent beads (# L4655, Ø 1µm – Sigma-Aldrich). Cells were then incubated for 5 min with propidium iodide to investigate their viability levels. Finally, the cells were detached in PBS for flow cytometry analysis.

#### 2.5.4 Cytokine analysis of supernatants

IL-6 secretion levels were measured in RAW264.7 supernatants by ELISA using a murine IL-6 kit (#00100438, Covalab), according to the furnished protocol. The absorbance was measured using the SpectroNano (BMG) and the data analyzed by the MARS software (BMG). Results are presented as mean +/- standard deviations of quadruplicate samples. The statistical analysis was performed according to the variance ratio (highest over lowest), with a Student (ratio < 4) or a Welch (ratio > 4) t-test and scored as follow: * p < 0.05; ** p < 0.01 and, *** p < 0.001.

#### 2.5.5 Endotoxin content

Endotoxin concentration of the HMGB1 samples used to stimulate macrophages was measured with a ToxiSensor kit (#L00350Y, #L00350, GenScript) according to the supplier’s instructions. Endotoxin concentrations of samples were between 0.7 - 1.5 ng/ml. Considering that HMGB1 samples were diluted 1:1000 and only 1 - 10 µl were added for stimulation, their LPS content was far too low to result in any macrophage stimulation, as was checked for control.

## 3 Results

Following the initial description by Yeo et al. that complement C1s can cleave HMGB1 (25), which we confirmed (24), a set of molecular analyses were designed to investigate and finely decipher the molecular details involved in this process, and some of its functional outcome.

### 3.1 HMGB1 cleavage by C1s is influenced by C1s molecular environment

In its serum physiological context, C1s is mainly found within the C1 complex, where it is associated to C1r and C1q. Therefore, we designed *in vitro* experiments aiming to compare HMGB1 cleavage using the same activated C1s molar concentration, either in the free state or within C1. As shown on **Figure 2**, HMGB1 cleavage is less effective when C1s is within C1, since the un-cleaved HMGB1 band remains stronger. Similar trends were obtained for all experiments performed at 5% and 10% E/S ratios (**Figure 2**), as well as in a preliminary test performed at a 40% E/S ratio (not shown). Since free C1s is more efficient, it was further used for the experiments aiming to decipher the C1s cleavage sites in HMGB1. As will be discussed later, differential enzymatic activity between free C1s and C1 on HMGB1 was among the criteria which raised our interest for this target while studying C1s variants identified in pEDS patients (24).

### 3.2 C1s primarily cleaves HMGB1 after K172, and secondarily after K65 and K127

**Figure 3:**
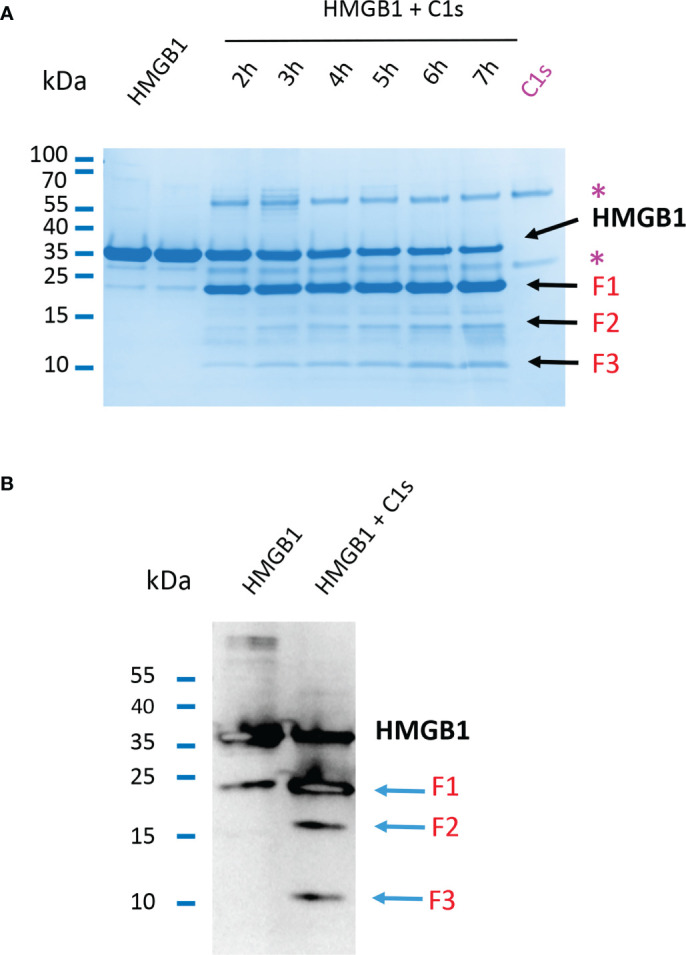
Primary and secondary C1s cleavage of HMGB1 lead to three N-terminal F1, F2 and F3 fragments. **(A)** 10 - 20% Tricine gel loaded with HMGB1 and HMGB1 + C1s incubated for 2 to 7 hours. Besides the main F1 fragment, which appears before 2 hours, two other bands (F2, F3) appear more lately and increase in intensity over time. Bands corresponding to C1s are labeled (*). **(B)** Western blot of HMGB1 and fragments incubated or not with C1s for 5 hours using an anti-HMGB1 antibody targeting its N-terminal 2 – 17 sequence. Three fragments F1 - F3 are obtained after digestion of HMGB1 (blue arrows). Specificity of the N-terminal antibody was verified separately (data not shown).

As shown above or previously (24), HMGB1 cleavage by C1s leads to one main primary digestion product, F1. Longer incubation times for the enzymatic digestion of HMGB1 by C1s yield also smaller fragments of about 10 to 15 kDa, as shown on **Figure 3A**, resulting from further cleavages. Using an antibody targeting the N-terminal sequence of HMGB1, western blot analysis clearly reveals three main N-terminal fragments that we named F1, F2 and F3, with apparent MW at about 20, 15 and 10 kDa, respectively (**Figure 3B**).

**Figure 4:**
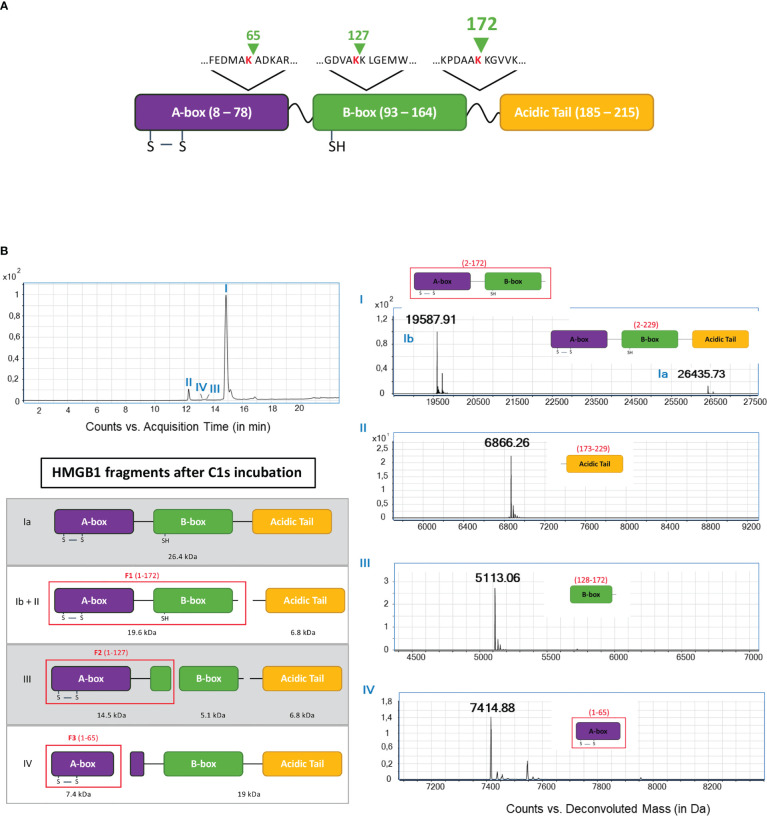
Fine LC/ESI mass spectroscopy analysis of HMGB1 incubated with C1s **(A)**. The cleavage sites deduced from the MS analysis are shown above the HMGB1 modular structure (green arrow shows where C1s cleavage occurs) **(B)**. The left MS panel shows an overview of the global spectrum with 4 peaks (I - IV). Peak I is subdivided into a main peak (Ib) at 19.6 kDa, corresponding to the mass of the 2-172 (F1) fragment, and a smaller peak (Ia) at 26.4 kDa, corresponding to undigested HMGB1. Peak II corresponds to the second product of the primary cleavage after K172, namely the C-terminal fragment (seq. 173 - 229) with a molecular weight of 6.8 kDa. Peak III comprises a fragment of the B-box (seq. 128 - 172) at 5.1 kDa. Peak IV corresponds to a fragment of the A-box (seq. 2 - 65) with a mass of 7.4 kDa. The left interpretation panel shows the attribution of the N-terminal fragments F1 - F3 within the HMGB1 frame: F1 corresponds to the sequence 1 - 172, F2 fits with the sequence 1 - 127 and F3 is the sequence 1 - 65.

Aiming to identify the fragments by mass spectrometry (MS), we used conditions leading to more complete HMGB1 digestion, mainly with longer incubation times (5 h). MS experiments and analyses of HMGB1/C1s liquid mix have been performed starting from the full-length HMGB1 (**Figure 4**), or from HMGB1 variants or fragments (next section). All these experiments resulted in redundant and consistent identification of three C1s cleavage sites in HMGB1. By showing a loss of 2 Da (mass of two hydrogens) as compared to the calculated mass, these MS analyses also consistently confirmed the presence of the disulfide bond in the A-box. As shown on **Figure 4**, the major peak I includes traces of un-cleaved HMGB1, plus a main fragment of 19.5 kDa, corresponding to the HMGB1 2-172 sequence (F1). The complementary 173-229 C-terminal fragment of 6.8 kDa is detected in the small peak II. These data clearly suggest that C1s mainly cleaves HMGB1 after K172.

MS detection and identification of the minor secondary cleavage products is quite difficult to achieve when starting from full-length HMGB1 (**Figure 4**). However, the small peak III reveals a 5.2 kDa fragment corresponding to the HMGB1 sequence from 128 to 172, suggesting cleavage after K127. Finally, peak IV reveals a 7.4 kDa fragment corresponding to the 2-65 fragment of HMGB1 (F3). Three C1s cleavage sites were thus identified by MS analyses of the HMGB1/C1s digestion mix, as summed up at the top of **Figure 4**.

### 3.3 The C1s cleavage site signature in HMGB1 contains atypical features

**Figure 5:**
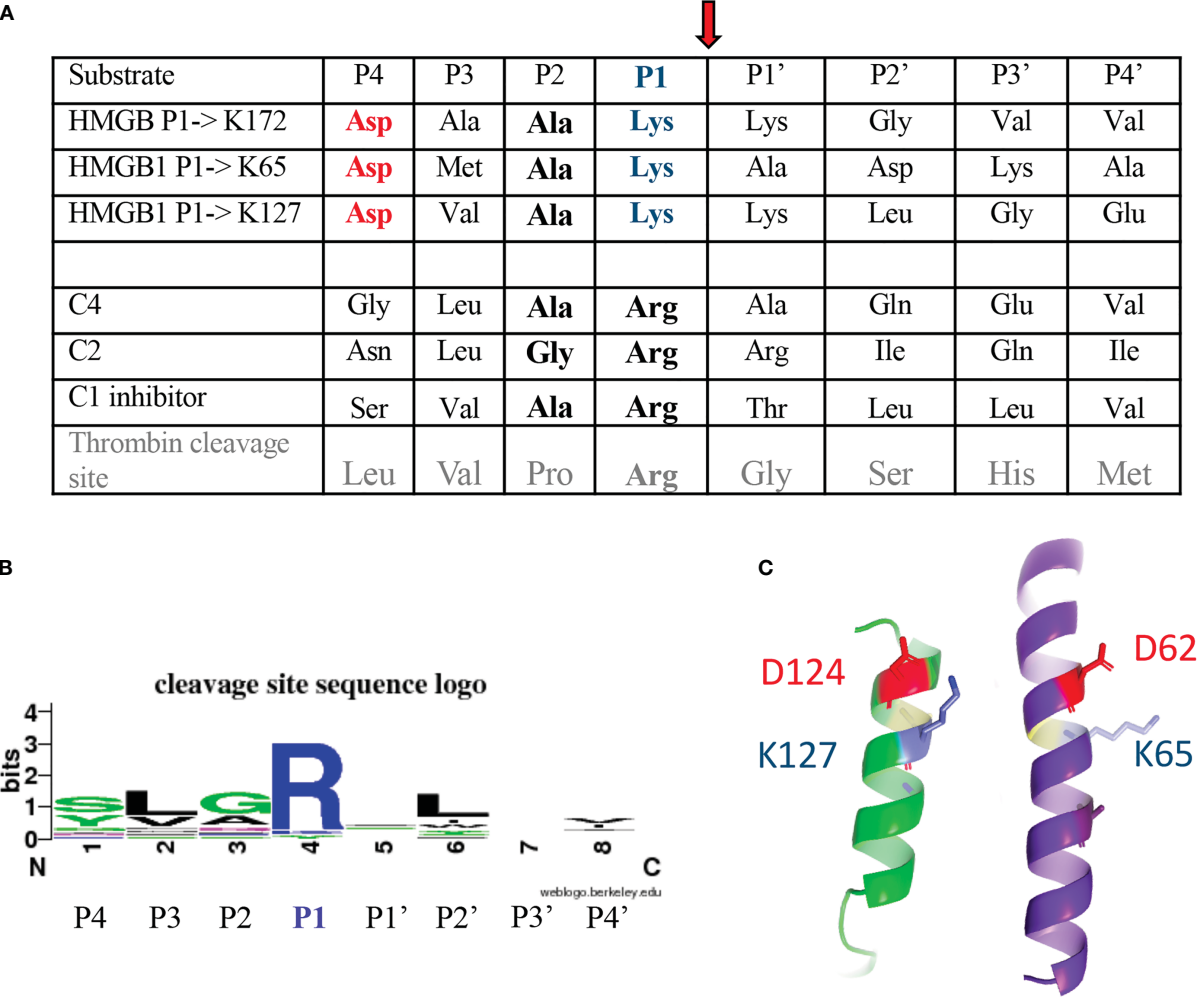
C1s cleavage sites in HMGB1 as compared to other targets. **(A)** Sequences upstream and downstream the C1s cleavage site are shown using the standard P4 to P4’ nomenclature, with cleavage of the scissile bond between P1 and P1’ residue (see red arrow). This terminology was used to define the extended interactions with different S4 to S4’ enzyme subsites of the P4 to P4’ sequence, these interactions defining the specificity of the proteases (43). The classical C1s targets are its two C4 and C2 substrates, leading to the activation of the complement system, as well as its regulator, C1-inhibitor. **(B)** The schematic cleavage site sequence logo found in the Merops database (25), based on 20 cleavages, is shown for comparison. The corresponding cleavage pattern is syg/Lv/GAq/R/-/L/-/vi, with a cleavage after arginine (R). **(C)** The cleavage sites newly identified in HMGB1 are found in helical contexts, this assumption being stronger for K65 and K127, and putative/transient for K172. In this configuration, the P4 aspartic acid residue (red) is close to the target P1 lysine (blue). This helical structure likely needs to unwind locally during cleavage.

As shown on **Figure 5A**, the three cleavage sites identified in HMGB1 show similar sequence features. They are characterized by a common cleavage after a lysine residue (in P1), preceded by a small alanine residue (in P2), a small hydrophobic residue (in P3), and an aspartic acid residue (in P4). This P1 to P4 terminology was used to define the extended interactions of the residues upstream (P4 to P1) and downstream (P1’ to P4’) the scissile bond with different S4 to S4’ enzyme subsites, these interactions defining the specificity of the proteases (43). When compared with the C1s cleavage sites previously identified for the classical targets of C1s, namely C4, C2 and C1-inhibitor, and more generally with the C1s cleavage site signature described in the Merops database (25), P1 Lys (instead of Arg) and P4 Asp appear as uncommon sequence features (**Figures 5A, B**). As will be discussed later on, these features become even more surprising when considering the structural context which would place the P4 Asp and P1 Lys in close proximity (**Figure 5C**), at least for the sites located within helices in the A- and B-boxes, after K65 and K127, respectively.

### 3.4 Confirmation of the cleavage data using HMGB1 variants

**Figure 6:**
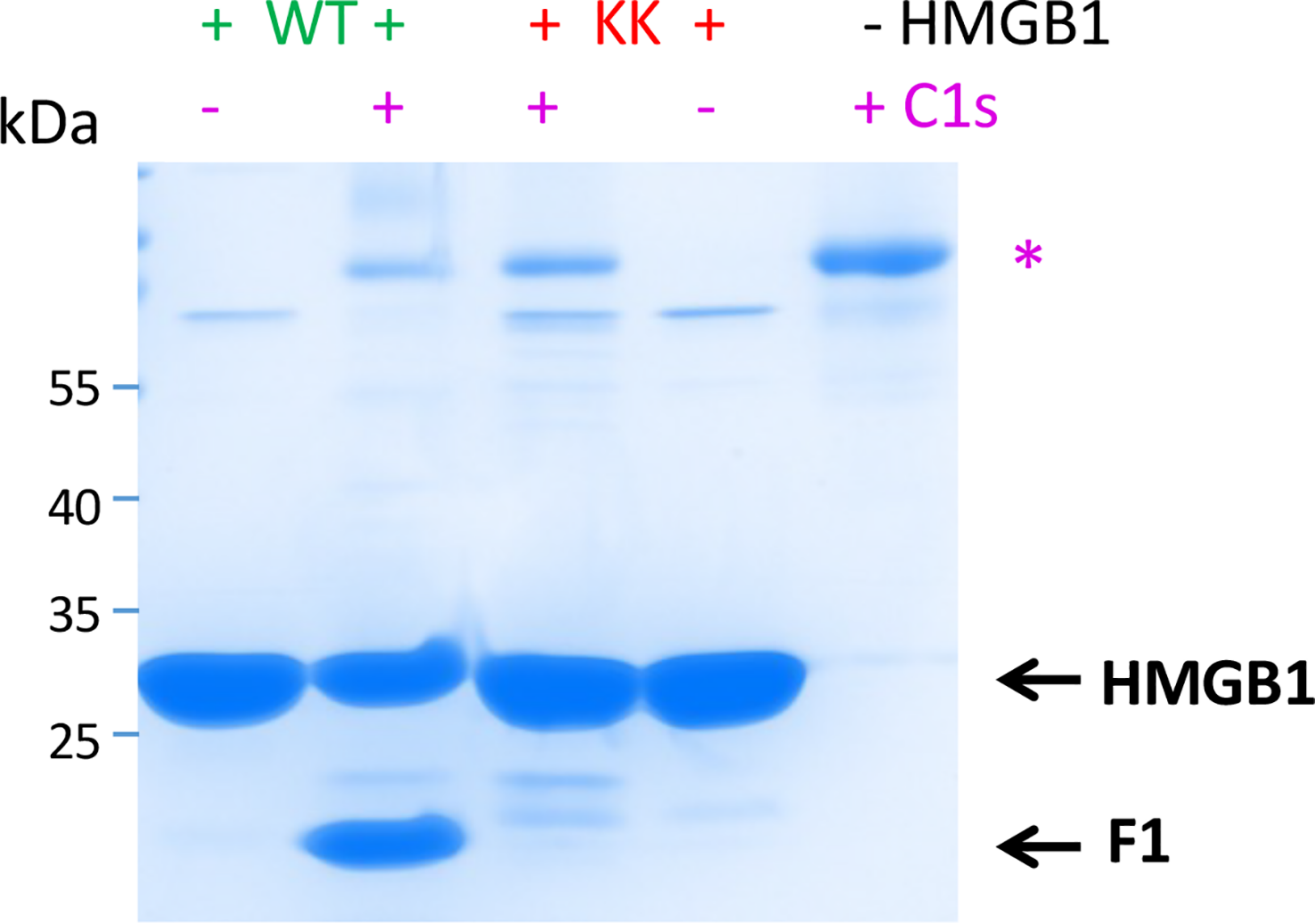
C1s primary cleaves HMGB1 after K172, as confirmed by K172E-K173G mutation. C1s cleaves HMGB1 WT far more efficiently than the KK variant carrying the K172E-K173G mutation. This is evidenced by the differential level of HMGB1 and primary cleavage product (F1, 1-172). Therefore, C1s primarily cleaves HMGB1 after K172. HMGB1 (6 μg) has been incubated for 100 min at 37°C, without (-) or with 5% (+) rC1s. SDS-PAGE analysis in non-reducing conditions. 2 μg rC1s have been deposited on the right lane for control. * as in **Figure 3**.

In order to confirm the identification of the primary cleavage site after K172, the double mutation K172E-K173G was introduced in HMGB1 to produce the HMGB1_KK mutant. All molecular details on HMGB1 variants are shown in **Figure S1** and **Table S1**. The double mutant has been designed because the initial MS data showed a minor trace of cleavage after K173. A double mutation was therefore chosen to avoid any possible compensatory cleavage after K173. As shown on **Figure 6**, the HMGB1_KK mutant gained almost complete resistance to C1s cleavage. This observation remains true even at 25% E/S ratio, without any apparition of F1 nor F2 fragment (**Figure S2**). However, a tiny amount of the F3 fragment was also detected in this context. We thus confirmed that K172 is the primary cleavage site for C1s in HMGB1.

**Figure 7:**
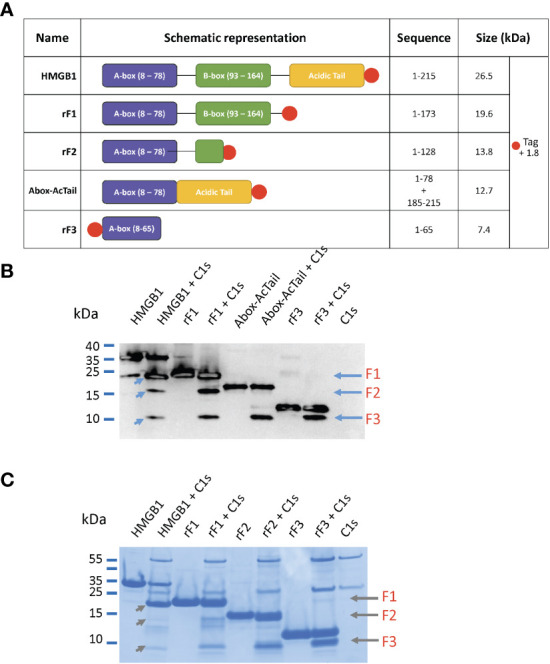
HMGB1 variants and their digestion by C1s. **(A)** List of the HMGB1 constructs used in this experiment with their corresponding sequence and molecular weight. The N- or C-terminal His-tag is shown with a red dot. **(B)** Western blot of HMGB1 and fragments incubated or not with C1s (at 10%) for 5 hours at 37°C using an N-terminal anti-HMGB1 antibody targeting the sequence 2 – 17 (as in **Figure 3**). The fragments loaded are: HMGB1, rF1(1-K173), Abox-AcTail, rF3(1-K65). The three fragments F1 - F3 can be found after digestion of HMGB1 (left blue arrows). F2 and F3 are present after rF1 cleavage and F3 only after cleavage of Abox-AcTail and rF3. Redundant fragment identification (as in **Figure 4**) was obtained using LC/ESI analyses performed on liquid mix of C1s with rF1, Abox-AcTail and rF3. This WB experiment was performed three times. **(C)** Same experiment as above analyzed by tricine gels, using rF2 instead of the Abox-AcTail.

The three N-terminal fragments, F1 to F3, have been recombinantly produced for further characterization, with a His-tag added for purification purpose (24), as detailed in section 2.2.1. These fragments were named accordingly rF1(1-K173), rF2(1-K128) and rF3(1-K65), and their modular structure is shown on **Figure 7A**. MS and WB analyses of the digestion of rF1 by C1s confirmed the release of the 2-127 fragment of 14.5 kDa (F2), the 128-172 fragment of 5.2 kDa, as well as the 2-65 fragment (F3) of 7.4 kDa (MS data not shown). MS and WB analyses of the digestion of rF3 by C1s also confirmed the release of a F3-like fragment (**Figure 7B**), after C1s cleavage of the N-terminal tag at the thrombin site (MS data not shown).

### 3.5 Focus on the common F3 fragment, which includes a large part of the A-box

Knowing that the HMGB1 A-box is a functional antagonist of HMGB1 and provides a potential regulatory feedback mechanism (27) (44), our study focused on N-terminal fragments, wondering if C1s cleavage could also generate N-terminal fragments with similar properties as the A-box. As shown on **Figure 7**, **S2**, the F3 fragment is a common product released by C1s cleavage of HMGB1, rF1, rF2 and rF3. Its presence was confirmed by several mass spectrometry analyses. Indeed, the 7.4 kDa N-terminal fragment was initially identified as the 2-65 sequence segment by MS analysis of a C1s digestion mix (5 h) of the Abox-AcTail HMGB1variant, where F3 is highly produced, as illustrated by the WB analysis shown in **Figure 7B**. In this HMGB1 variant, the A-box is fused to the acidic tail (**Figure 7A**). This artificial construct was inspired by a publication where similar constructions were shown to enhance A-box antagonist properties (44). As F3 includes a large part of the A-box, it might regulate HMGB1 function, as will be evaluated in the next section.

### 3.6 Anti-inflammatory properties of the recombinant F3 fragment as compared to the A-box and HMGB1

**Figure 8:**
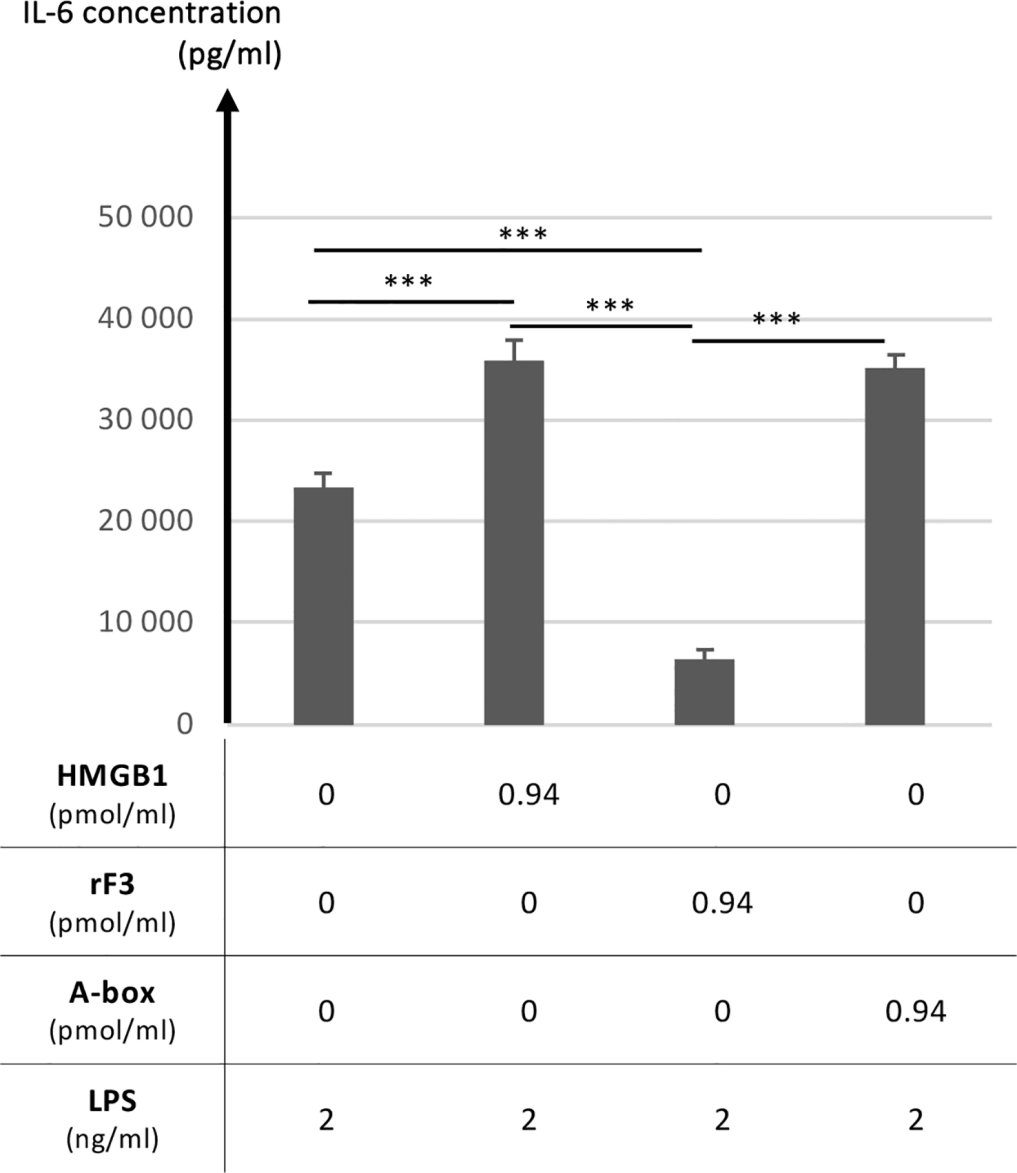
Anti-inflammatory properties of the rF3 fragment as compared to the A-box and HMGB1. The secretion of the inflammatory IL-6 cytokine is compared after stimulation by LPS at 1 or 2 ng/ml, alone or preincubated with HMGB1, rF3 or the A-box (see experimental details in section 2.5). A strong and significant reduction of IL-6 secretion is observed with rF3, as compared to LPS alone and in complex with HMGB1 and the A-box. Results are presented as mean +/- standard deviations (n=4). Control experiments showed the absence of IL-6 secretion in absence of LPS (**Figure S3**) and complementary experiments further illustrate the anti-inflammatory effect of rF3 as compared to the A-box (**Figure S4**). This data on rF3 is representative of two independent replicates performed at different concentrations. (***, p<0.001)

The functional comparison between rF3 fragment (1-K65), HMGB1 and A-box was thus explored regarding the modulation of the secretion of the inflammatory IL-6 cytokine following stimulation with low concentrations of LPS (25, 45). This question was addressed using RAW264.7 cells stimulated with LPS alone or in complex with HMGB1 or its fragments. This latter experimental setting using preincubated complexes has been previously introduced by others to obtain significant and reproducible results to analyze the synergistic amplification of inflammatory signaling of LPS (or other TLR ligands) by HMGB1 (9). Used alone, and not in complex with LPS, neither HMGB1 nor its fragments induced inflammatory IL-6 secretion, whereas HMGB1 in complex with LPS enhanced IL-6 secretion (**Figure S3**). As shown on **Figure 8**, a strong and significant reduction of IL-6 secretion with rF3 was reproducibly observed, as compared to LPS alone or in complex with the A-box (**Figure S4**). Increased secretion of IL-6 with HMGB1 and the A-box complexed to LPS is consistent with previous observations (9). These experiments show a strong anti-inflammatory effect of rF3, as compared to the A-box or to LPS alone, which was unexpected.

### 3.7 Impact of the disulfide bond in HMGB1 on F3 production by C1s

**Figure 9:**
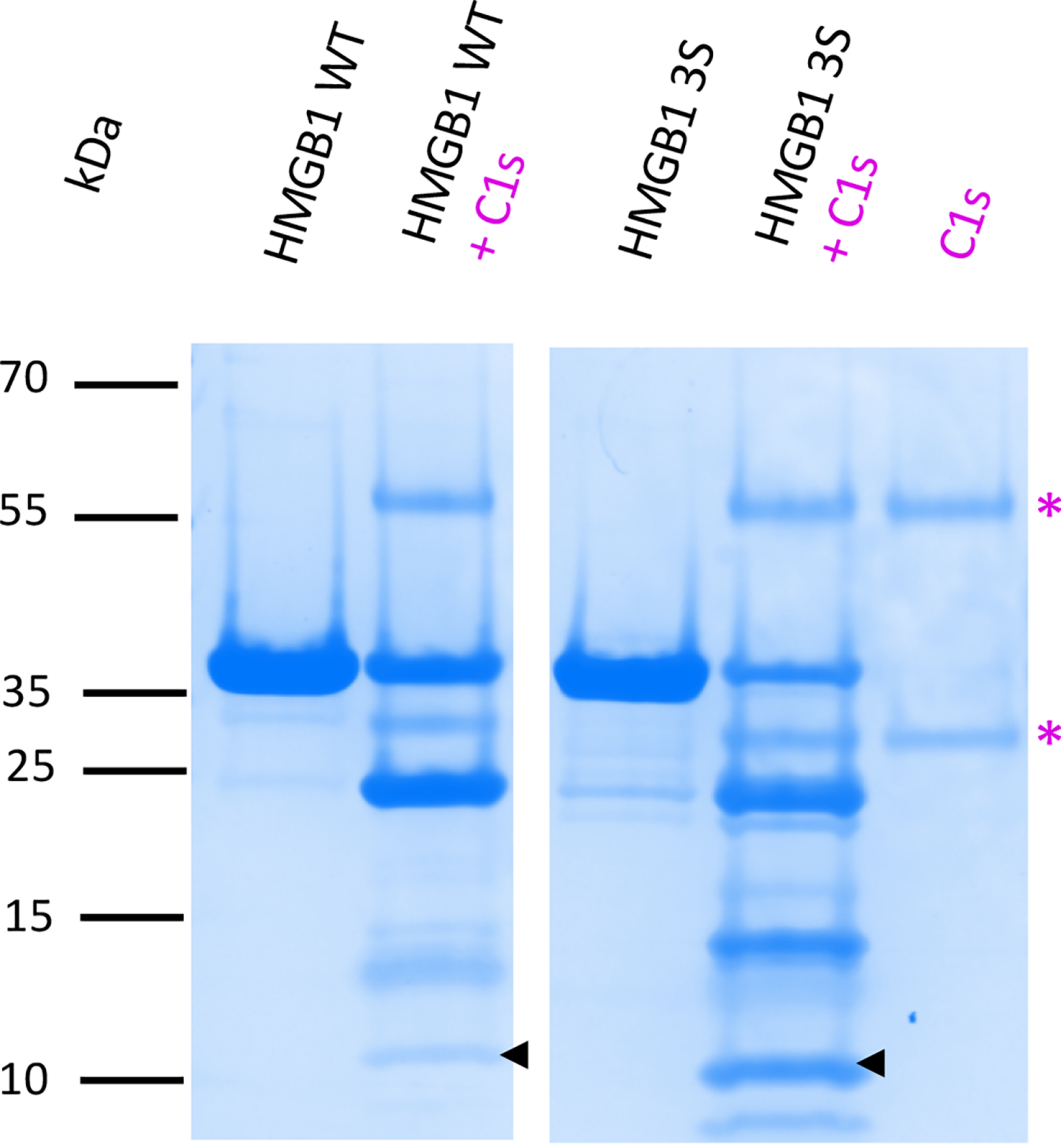
C1s cleavage is more efficient in absence of the disulfide bridge in HMGB1. This comparative digestion experiment shows that C1s digests more effectively the 3S variant (where all cysteines are replaced by a serine) than HMGB1. As shown by the arrows, more F3 fragment is released from C1s cleavage of the 3S variant. The 10-20% Tricine gel shown for the C1s digestion is representative of two experiments. 5 μg HMGB1 or 3S were digested at 10% E/S for 90 min at 37°C. To consolidate the view of a more effective digestion of the 3S variant with more F3 released, a complementary experiment performed with increasing incubation times (from 1 to 7 h) is shown in **Figure S5**. * as in **Figure 3**.

Considering the functional interest of the F3 fragment, we started to investigate the question of a possible impact of HMGB1 molecular environment on C1s digestion outcome. As mentioned previously, the purified recombinant HMGB1 WT and its fragments contain a disulfide bridge in the A-box. What could be the impact of the disulfide bridge in the A-box regarding C1s digestion? To investigate this question, a 3S HMGB1 variant was produced, where all cysteines were mutated into serine (**Figure S1**). As illustrated in **Figure 9**, **S5**, this 3S variant is cleaved more effectively than the WT, yielding more F3 fragments.

### 3.8 C1s as compared to other proteases in cleaving HMGB1

**Figure 10:**
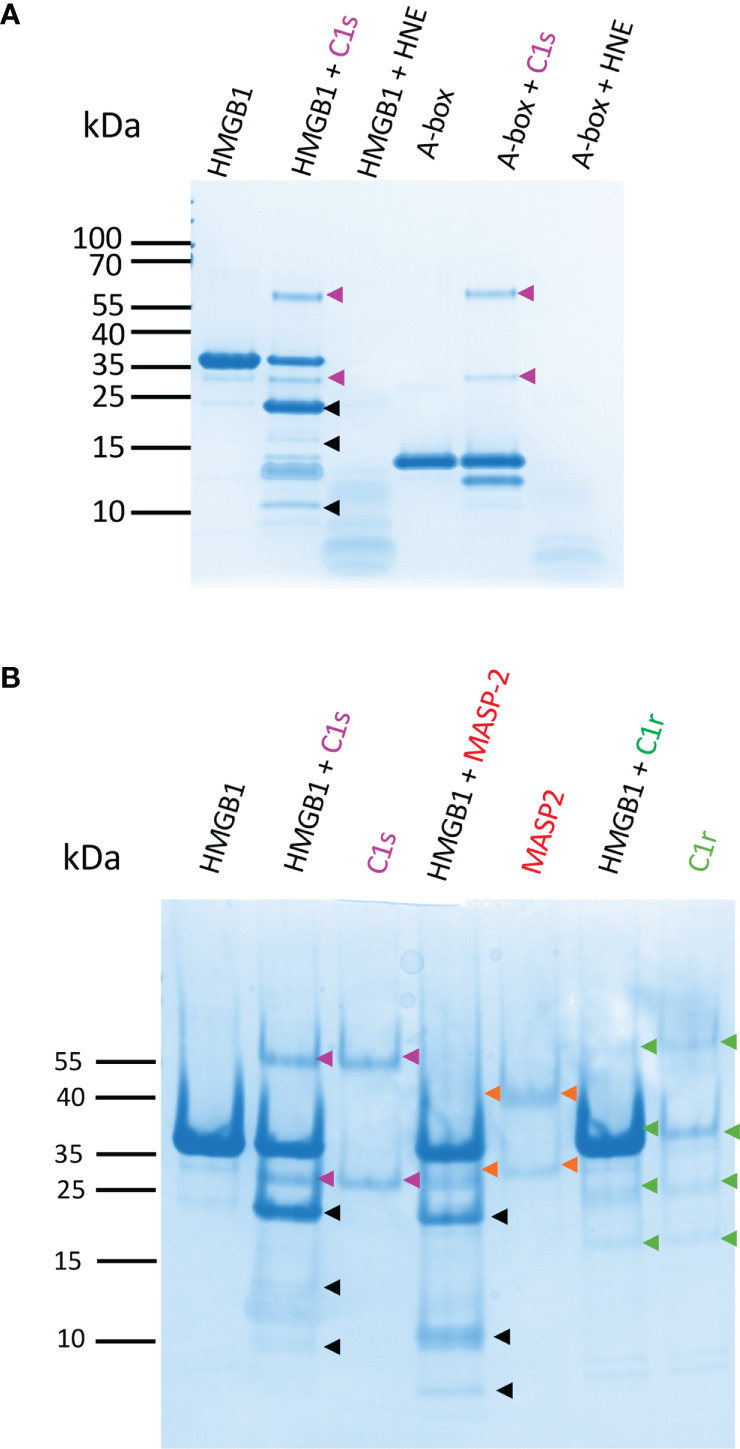
HMGB1 is mildly digested by C1s and MASP-2 whereas HNE degrades it rapidly into numerous small molecular weight peptides. **(A)** 10 - 20% Tricine gels loaded with HMGB1 or the A-box incubated with C1s or HNE for 90 min. HNE cleavage of HMGB1 yields many undefined small molecular weight fragments and completely digests HMGB1. The A-box is not cleaved by C1s (as identified by LC/ESI MS, C1s only cleaves the tag off after 5 h, as seen in **Figure 6B** for rF3). In contrast, HNE rapidly degrades the A-box into small pieces. Gels were loaded with 0.1 nmol of HMGB1 (3 µg) or A-box (1.35 µg). HMGB1 and C1s or HNE were mixed at a 10% E/S molar ratio in TBS. A gel representative from two experiments is shown. HNE bands are not seen in these conditions, suggesting autolysis. **(B)** 10-20% Tricine gels loaded with HMGB1 incubated with C1s, MASP-2 or C1r for 90 min at 10% E/S. C1r does not cleave HMGB1. MASP-2 cleaves HMGB1 quite similarly as C1s, but the secondary cleavage products and proportions differ. A gel representative from two experiments is shown.

For the comparison between C1s and other enzymes, HMGB1 was cleaved using the same molar ratio and incubation conditions. C1s was compared to human neutrophil elastase (HNE). HNE is indeed present in inflammatory conditions where neutrophils are abundant (22). In this case, the proteolytic profile obtained with HNE was strikingly different (**Figure 10A**). Indeed, C1s digestion of HMGB1 was by far slower, restricted and specific when compared to HNE in the same conditions. After 90 min, HNE has completely degraded HMGB1 as well as the isolated A-box. When we tried to identify HNE-derived fragments using longer incubation times, as we have done for C1s, no fragment identifiable by MS remained after HNE digestion. Intriguingly, the isolated A-box resisted to C1s cleavage, although it includes a C1s cleavage site (**Figure 10A**). The fact that C1s did not digest the isolated A-box suggests that the presence of the B-box or AcTail is necessary for C1s to cleave after K65.

C1s was further compared with two homologous complement proteases, C1r and MASP-2 (which activates the complement lectin pathway). MASP-2 shares the same specificity as C1s in terms of complement targets, namely C4 and C2, whereas C1r only activates C1s. As expected, **Figure 10B** shows that the C1r protease did not cleave HMGB1, but the complement MASP-2 did. There were some subtle differences between C1s and MASP-2 cleavage outcome. For example, less F1 and F2, and more F3 were obtained with MASP-2, as well as an additional small fragment below 10 kDa.

## 4 Discussion

Vertebrate immune defense and homeostasis rely on several alarm systems and their effective crosstalk. The canonical function of the complement C1s protease is the activation of the classical complement pathway within the C1 complex. However, the pathological impact of *C1S* genetic deficiencies, such as severe lupus or periodontal EDS, cannot be simply explained only by considering this function, therefore suggesting auxiliary non-canonical functions for this protease. A crosstalk between the HMGB1 alarmin and the complement system components was recently evidenced at different levels (46, 47), which includes the C1s auxiliary enzymatic activity on HMGB1 (16). This enzymatic activity is modulated under several conditions. For example, C1s cleaves more efficiently HMGB1 when it is free, as compared to C1s within the C1 complex (**Figure 2**). In normal human serum, C1s is mainly observed within the C1 complex, in a proenzyme form, and HMGB1 is absent, only seen in pathogenic/inflammatory contexts. Moreover, C1 inhibitor (C1-INH) is present, and we have previously shown that C1-INH inhibits HMGB1 cleavage by C1s (24). Thus, HMGB1 will not be cleaved by C1s in normal human serum. However, our observations will be relevant to pathological situations, when C1s gets activated independently of C1q, or when active C1s is out of C1-INH mediated control. This study therefore focuses on how free complement C1s protease cleaves HMGB1 and how this could impact HMGB1 inflammatory cytokine signaling. This is interesting because activated C1s is found in some specific disease contexts such as SLE (48) but also in other autoimmune and cancer disease contexts (49–51). At present, it is not known if this pathological activated C1s is in a free form or not, but it likely escapes from the functional control of C1q and C1-INH. Moreover, it has been shown in the very rare periodontal EDS syndrome (pEDS) that C1R or C1S mutations lead to a gain of function for C1s, which becomes constitutively activated independently of C1q (24, 52). However, the molecular mechanism leading to periodontitis-like symptoms and tooth loss remain to be deciphered. Recent advances in the field of this rare pEDS syndrome identified altered cytokine secretion in monocytes (53), as well as C1s-mediated collagen cleavage in fibroblasts (54), although this latter accessory C1s enzyme activity cannot be observed *in vitro* at normal room temperature.

Three C1s cleavage sites in HMGB1 were experimentally deciphered (**Figures 3**, **4**). The main site is after K172 (**Figure 6**); two secondary sites are after K65 and K127 (**Figures 1**–**4**). Incidentally, thanks to the use of tricine gels which allow better separation of low molecular weight species, we also observed partial C1s cleavage at the thrombin site introduced at the N-terminus of the A-box and rF3 constructs (**Figures 5**, **7**, **10**, **S1**). These results are fully consistent with the known preference for trypsin-like proteases to cleave after an arginine or a lysine residue at the P1 site, according to the Schechter and Berger nomenclature (43). However, when compared to previously identified C1s cleavage sites, the P4-P1 aspartic acid-lysine dyad in HMGB1 is clearly uncommon (**Figure 5**). This can explain why C1s cleavage sites were not correctly predicted by Yeo et al. (25). It also explains why complementary experiments have been performed in this study to confirm these cleavage site data. Intriguingly, the helical context of the C1s cleavage sites in the A- and B-box (**Figure 5C**) is in strong contradiction with the known serine proteases conserved feature which is to cleave their target within loops or strands. Indeed, for most serine protease/target complexes, the residues from P3 to P2’ are observed in an extended or strand conformation. It has been proposed therefore that helical or turn-like conformations would be too large to occupy the restricted space in the enzyme active site (55, 56). The unexpected helical context in HMGB1 boxes would therefore suggest that local unfolding/unwinding might occur before C1s cleavage at these positions. This would fit with the experimental observations that C1s cleavage of HMGB1 is quite slow, especially in comparison to HNE (**Figure 10A**) or other inflammatory proteases (22). This hypothesis would also be consistent with the observation that K172 is the primary cleavage site. Indeed, K172 and K173 residues are in a linker area predicted as flexible. This hypothesis would also fit with the observation that HMGB1 cleavage by C1s is enhanced in absence of the disulfide bridge in the A-box, as seen for the cleavage of the 3S variant (**Figures 9**, **S5**). Conversely, the observation that the A-box, as well as the B-box (not shown) are not cleaved by C1s (**Figure 10A**) also suggests that extended interactions are needed to locally unwind the helical structure around the target bond to be cleaved. These extended interactions would be present in HMGB1, rF1, rF2 and Abox-AcTail, which release F3 upon C1s cleavage.

**Figure 11:**
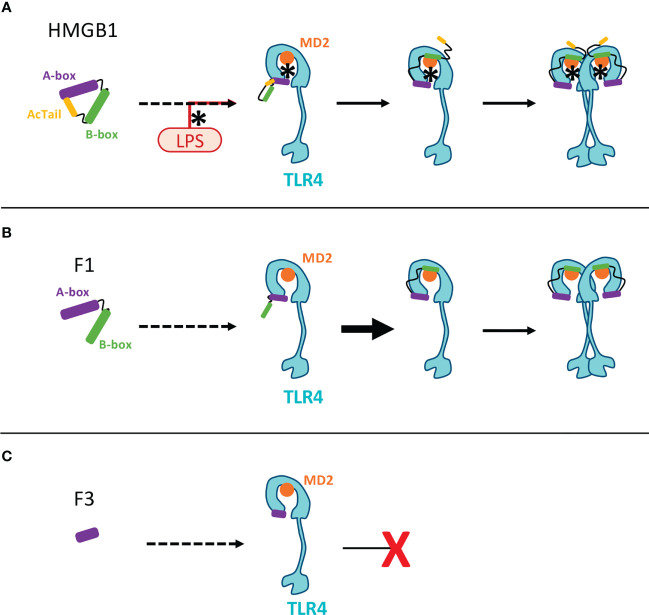
Molecular hypotheses associated to HMGB1 binding toTLR4-MD2 and the inhibiting action of the F3 fragment. This scheme aims to illustrate previous working hypotheses published by others on the opposite effects of the A- and B-box fragments on TLR4-mediated signaling (28). These hypotheses open clues to understand the possible impact of HMGB1 fragments produced by C1s. **(A)** In the full length HMGB1, the AcTail dynamically binds to the HMGB1 boxes, thereby regulating the binding properties of HMGB1 (58). LPS binding to HMGB1 (using pre-incubation in our conditions, as in (9)) counteracts the AcTail mediated inhibitory effect. Three successive steps have been previously proposed for HMGB1 binding to the TLR4/MD2 complex (59): 1) the A-box first binds to TLR4 through R24, K28 and the tip of the first helix of HMGB1; 2) then the B-box can bind MD2; 3) The B-box then mediates TLR4 dimerization and signaling. **(B)** The lack of the AcTail in the F1 fragment likely enhances binding to DNA and to the TLR4/MD2 complex, as shown for other fragments similarly lacking the AcTail (60). In this case, LPS may not be necessary for binding. The same successive binding steps starting from the binding of the A-box to TLR4, as described above, can take place (59). **(C)** As already proposed for the A-box, a tight binding of the F3 fragment to TLR4 could prevent the binding of HMGB1 by competitive inhibition (59). The binding of this F3 fragment to TLR4 does not induce dimerization (because of the lack of B-box) and thus no signaling. The only molecular difference between the F3 and A-box fragments is the extremity after K65, present in the A-box but missing in the F3 fragment. This part, corresponding to the end of helix 3, protrudes from the core formed by the association of the three helices (**Figure 1)**. It is not part of the binding site for TLR4/MD2 (59), and binding to TLR4 might be more stable without this part.

The comparative functional analysis has been focused on the F3 fragment which is progressively released during C1s digestion (**Figure 3**). F3 represents indeed a common ‘final’ C1s digestion product on the N-terminal side of HMGB1, which is released from various constructs (**Figure 7**). The molecular environment of HMGB1 may of course impact the release of F3 by C1s, as illustrated by some preliminary *in vitro* experiments. Of note, F3 is more efficiently released by C1s in absence of disulfide bridge in HMGB1 (**Figure 9**), as mentioned above, and thus potentially in contexts where HMGB1 is in fully reduced form. Indeed, in our *in vitro* setting, F3 is already clearly seen at 60 or 90 minutes in absence of disulfide bridge (**Figures 9**, **S5**), whereas F2 and F3 fragments are observed mainly after 2 h incubation in presence of the disulfide bridge (**Figure 3**). In addition, when considering that lysine acetylation could modulate C1s cleavage *in vivo*, it is relevant to notice that K65 was reported to never be acetylated (57), meaning that the release of the F3 fragment is not compromised by lysine acetylation. F3 includes a large part of the A-box. Knowing the antagonist role of the recombinant A-box in many functional contexts, we wondered if the F3 fragment could represent a natural antagonist released by C1s cleavage. This would provide a molecular clue to the previous observation that overnight digestion of HMGB1 by C1s strongly reduced the amplification of IL-6 secretion following moderate LPS activation (25). And indeed, the F3 fragment showed a potent anti-inflammatory effect, which was not the case for the A-box in the experimental setting where it is preincubated with LPS (**Figure 8**). As mentioned before, this protocol from Hreggvidsdottir et al. (9) was chosen because it allows more reproducible and significant observations. In this protocol, IL-6 secretion very likely depends on TLR4 (9). Such a difference in responses between rF3 and A-box, two fragments which only differ by a C-terminal 19 amino-acids deletion in rF3, was an unexpected outcome of this study. This observation can be interpreted considering the current hypothesis that the A-box antagonist effect is obtained through competitive-binding to TLR4 (**Figure 11**). The current hypothesis is that such binding does not induce TLR4 dimerization (and thus signaling), but prevents the binding of HMGB1. On the opposite side, the isolated B-box is an agonist because it binds to the TLR4/MD2 interface and induces dimerization, and thus signaling (28, 59). Interestingly, the F3 fragment includes all the essential TLR4-binding domain identified in the A-box, namely R24, K28 and the tip of helix 1 (59). Looking back at the structure (**Figure 1**), we may hypothesize that cleavage after K65 will remove a part which partly interferes with TLR4 binding, enhancing the accessibility of the TLR4 binding sites. Thus, this result suggests a significant functional impact of C1s cleavage in the A-box (**Figure 11**).

In contrast to C1s, the major inflammatory proteases were shown to degrade the HMGB1 A-box, as shown for comparison with HNE in **Figure 10A**. Similar results were previously shown for HNE, cathepsin G or the matrix metalloproteinase 3 (22). The cleavage and release of the C-terminal acidic tail is often the first processing step, including for C1s (**Figure 6**). Because of the regulatory role of the C tail, its absence in the F1 fragment implies dysregulated HMGB1 functions. For example, when the second nuclear localization signal of HMGB1 (NLS2, residues 179-185) is missing, the way back to the nucleus is closed (3). Because the acidic DE repeats in the acidic tail are involved in dynamic autoinhibition of HMGB1 in the free state, their removal promotes some binding activities of HMGB1 (60). This is true for charged ligands such as DNA, because intramolecular interactions of the DE repeats with the DNA-binding A- or B-boxes and their intermolecular interaction with DNA are mutually exclusive (58, 61). In the same line, removal of the C-terminal acidic tail has been shown to enhance HMGB1 binding affinity for the TLR4/MD-2 complex in absence of LPS *in vitro* (60), while retaining similar TNF inflammatory signaling properties (27). The same hypothesis may apply to TLR2 binding (62).

All these studies reveal the potential of some proteases to regulate the function of HMGB1. As another example, the thrombin/thrombomodulin complex cleaves off the 17 N-terminal residues of HMGB1, a process which also reduced HMGB1 inflammatory role, as measured by TNF-α and iNOS mRNA induction and TNF-α secretion in RAW 264.7 cells (63). Looking at the complement proteases homologous to C1s, we confirmed that C1r has a different and highly restricted substrate specificity (64), and therefore does not cleave HMGB1, while MASP-2, which cleaves complement C4 and C2, also cleaves HMGB1, likely at the same cleavage sites (**Figure 10B**). However, MASP-2 is far less abundant than C1s in serum. To our knowledge, the only known unrelated enzyme which can release a N-terminal HMGB1 fragment quite similar to F3 is the intracellular caspase 1 (13). Indeed, HMGB1 cleavage after D67 by caspase-1 was shown to be essential for its regulatory role on immune tolerance for apoptotic cells mediated by dendritic cells (13). *In vivo*, the recombinant A-box, but also caspase-1 cleavage of HMGB1 have been shown to be protective against sepsis, a property depending on RAGE (13). This is related to the immunomodulatory property of the A-box (and its 1-67 fragment) to reverse the tolerance induced by apoptotic cells, which prevents immune defense against a secondary infection (*e.g.* by *Candida albicans*).

In conclusion, this and previous studies highlight the impressive modularity of HMGB1 functions, which depend on its location and can be finely tuned by the oxidative or proteolytic context. Thus, by switching between different states, HMGB1 can orchestrate different steps spanning from leucocyte recruitment, inflammation signaling and resolution, as well as tissue repair (65), which are essential for immune defense and proper healing. This study provides new insights on how proteolytic cleavage of HMGB1 by C1s can modulate the function of HMGB1. The smallest N-terminal F3 fragment could provide a potent brake for the inflammatory process, opening the way to dampen inflammation. The more efficient cleavage of HMGB1 in absence of disulfide bridge suggests that C1s cleavage might be more efficient in defined cellular and tissue contexts, where HMGB1 is in fully reduced form. This study is likely relevant to some pathological contexts, where both HMGB1 and complement play a role (16), such as autoimmune, cancer, or neurodegenerative diseases, although this will need further confirmation. For example, beneficial preclinical effects by box A therapy was first reported in experimental arthritis (66) and activated C1s has been observed in the context of rheumatoid arthritis, both in fluids and tissues (50, 67). Further studies may examine the therapeutic potential of F3-like fragments which reduce cytokine secretion, for example in sepsis (13, 27) or other pathological context where box A therapy has shown beneficial preclinical effects, such as transplantation, ischemia-reperfusion injury, acute lung injury, acute liver failure or stroke (8, 68). Other promising HMGB1 variants which can release the F3 fragment by C1s cleavage, as shown in this study, are the Abox-AcTail (44) and the HMGB1 3S variant (69–71).

## Data availability statement

The original contributions presented in the study are included in the article/**Supplementary Material**. Further inquiries can be directed to the corresponding author.

## Author contributions

ML and JK performed biochemical and cellular experiments and discussed the data. AC, IB and FD performed protein engineering and production, and initial biochemical experiments. LS performed MS experiment and their detailed analysis to precisely identify C1s cleavage sites. JV engineered the 3S mutant and the B-box fragment and analyzed their cleavage by C1s. TR and BD conceived the experiments on RAW264.7 cells and helped in their interpretation. NT supervised some protein productions and C1 cleavage experiments. VR and CG conceived the biochemical experiments and discussed the data. CG prepared the original manuscript draft and worked at the structural interpretation. All authors contributed to the article and approved the submitted version.

## Glossary

**Table:**

C1	complement component 1 (C1q, C1r, C1s sub-units)
C1-INH	C1 inhibitor
C2	complement component 2
C4	complement component 4
C23	cysteine 23
C45	cysteine 45
C106	cysteine 106
CP	classical complement pathway
DAMPs	damage-associated molecular patterns
DE	aspartic and glutamic acids
DNA	deoxyribonucleic acid
E/S	enzyme by substrate ratio
EDS	Ehlers-Danlos syndromes
ELISA	enzyme-linked immunosorbent assay
F1	fragment 1
F2	fragment 2
F3	fragment 3
FBS	fetal bovine serum
FPLC	fast protein liquid chromatography
HEK293-F cells	human embryonic kidney 293 Fast-growing cells
His	histidine
HMGB1	high-mobility group box 1
HPLC	high pressure liquid chromatography
HNE	human neutrophil elastase
IL-6	interleukin 6
ITPG	isopropyl β-d-1-thiogalactopyranoside
K65	lysine 65
K128	lysine 128
K172	lysine 172
K172E-K173G	lysine 172 mutated in glutamic acid and lysine 173 in glycine
LC/ESI-MS	liquid chromatography electrospray ionization mass spectrometry
LPS	lipopolysaccharide
MASP-2	mannan-binding lectin associated serine protease 2
MD2	myeloid differentiation protein 2
MS	mass spectrometry
MW	molecular weight
Nter	N-terminal
PBS	phosphate buffer saline
pEDS	periodontal Ehlers-Danlos syndromes
R24	arginine 24
RAGE	receptor for advanced glycation end products
rF1	recombinant fragment 1
rF2	recombinant fragment 2
rF3	recombinant fragment 3
RPMI	Roswell Park Memorial Institute medium
S. pneumoniae	Streptococcus pneunomiae
SDS-PAGE	sodium dodecyl sulfate polyacrylamide gel electrophoresis
SLE	systemic lupus erythematosus
TBS	tris buffer saline
TFA	trifluoroacetic acid
TLR2	toll-like receptor 2
TLR4	toll-like receptor 4
TLR	toll-like receptors
TNF	tumor necrosis factors
TNFα	tumor necrosis factor alpha
w/v	weight by volume ratio
WB	western blot
WT	wild type
