# Supplementary material for: HMGB1 cleavage by complement C1s and its potent anti-inflammatory product
Source: Front Immunol. 2023 Apr 26;14:1151731. doi: 10.3389/fimmu.2023.1151731 (PMC10169756; doi:10.3389/fimmu.2023.1151731)

Figure S1. Sequences of HMGB1 and its variants

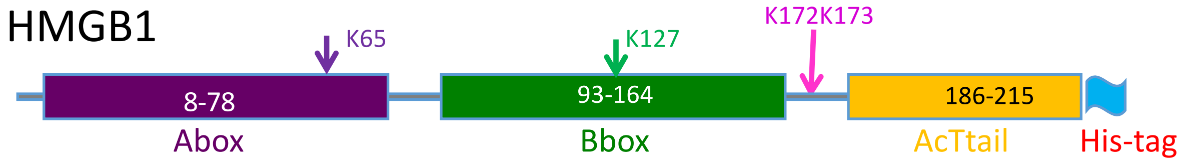

HMGB1: * Plasmid initially described in: *Nativel B, Marimoutou M, Thon-Hon VG, Gunasekaran MK, Andries J, Stanislas G, et al. Soluble HMGB1 is a novel adipokine stimulating IL-6 secretion through RAGE receptor in SW872 preadipocyte cell line: contribution to chronic inflammation in fat tissue. PLoS ONE. (2013) 8:e76039. doi: 10.1371/journal.pone.0076039*

MGKGDPKKPRGKMSSYAFFVQTCREEHKKKHPDASVNFSEFSKKCSERWKTMSAKEKGKFEDMAKADKARYER
EMKTYIPPKGETKKKFKDPNAPKRPPSAFFLFCSEYRPKIKGEHPGLSIGDVAKKLGEMWNNTAADDKQPYEK
KAAKLKEKYEKDIAAYRAKGKPDAAKKGVVKAEKSKKKKEEEEDEEDEEDEEEEEDEEDEDEEEDDDDELVPR
GSLEHHHHHH*

HMGB1-KK

MGKGDPKKPRGKMSSYAFFVQTCREEHKKKHPDASVNFSEFSKKCSERWKTMSAKEKGKFEDMAKADKARYER
EMKTYIPPKGETKKKFKDPNAPKRPPSAFFLFCSEYRPKIKGEHPGLSIGDVAKKLGEMWNNTAADDKQPYEK
KAAKLKEKYEKDIAAYRAKGKPDAAEGGVVKAEKSKKKKEEEEDEEDEEDEEEEEDEEDEDEEEDDDDELVPR
GSLEHHHHHH

rF1(1-K173)

MGKGDPKKPRGKMSSYAFFVQTCREEHKKKHPDASVNFSEFSKKCSERWKTMSAKEKGKFEDMAKADKARYER
EMKTYIPPKGETKKKFKDPNAPKRPPSAFFLFCSEYRPKIKGEHPGLSIGDVAKKLGEMWNNTAADDKQPYEK
KAAKLKEKYEKDIAAYRAKGKPDAAKKGHHHHHH

B-box

MGNAPKRPPSAFFLFCSEYRPKIKGEHPGLSIGDVAKKLGEMWNNTAADDKQPYEKKAAKLKEKYEKDIAAYR
AGHHHHHH

rF2(1-K128)

MGKGDPKKPRGKMSSYAFFVQTCREEHKKKHPDASVNFSEFSKKCSERWKTMSAKEKGKFEDMAKADKARYER
EMKTYIPPKGETKKKFKDPNAPKRPPSAFFLFCSEYRPKIKGEHPGLSIGDVAKKGHHHHHH

Abox-AcTail

MGKGDPKKPRGKMSSYAFFVQTCREEHKKKHPDASVNFSEFSKKCSERWKTMSAKEKGKFEDMAKADKARYER
EMKTYEEEEDEEDEEDEEEEEDEEDEDEEEDDDDELVPRGSLEHHHHHH

Abox

MGSSHHHHHHSSGLVPRGSHMGKGDPKKPRGKMSSYAFFVQTCREEHKKKHPDASVNFSEFSKKCSERWKTMS
AKEKGKFEDMAKADKARYEREMKTYIPPKGE

rF3(1-K65)

MGSSHHHHHHSSGLVPRGSHMGKGDPKKPRGKMSSYAFFVQTCREEHKKKHPDASVNFSEFSKKCSERWKTMS
AKEKGKFEDMAK

HMGB1 3S

MGKGDPKKPRGKMSSYAFFVQTSREEHKKKHPDASVNFSEFSKKSSERWKTMSAKEKGKFEDMAKADKARYER
EMKTYIPPKGETKKKFKDPNAPKRPPSAFFLFSSEYRPKIKGEHPGLSIGDVAKKLGEMWNNTAADDKQPYEK
KAAKLKEKYEKDIAAYRAKGKPDAAKKGVVKAEKSKKKKEEEEDEEDEEDEEEEEDEEDEDEEEDDDDELVPR
GSLEHHHHHH

Table S1. HMGB1 WT and variants nomenclature with corresponding parameters used to estimate protein concentration.

HMGB1 WT and variants	Name	Mw (Da)	Abs (1%, 1 cm) at 280 nm
HMGB1 1-215	WT	26,568	0.811
HMGB1 1-173	rF1	20,728	1.034
HMGB1 1-128	rF2	15,634	0.733
HMGB1 1-65	rF3	9,711	0.720
HMGB1 A-box 1-84	A-box	11,975	0.843
HMGB1 B-box 93-164	B-box	11,554	0.992
HMGB1 C23S C45S C106S	3S	26,520	0.808
HMGB1 K172E K173G	KK	26,498	0.809
HMGB1 1-65/186-215	Abox-AcTail	14,598	0.683

Table S2. Proteases and corresponding parameters used to estimate protease concentration

Protease	Mw (Da)	Abs (1%, 1 cm) at 280 nm
C1s (plasma)	79,800	1.45
rC1s (with Flag tag)	80,665	1.52
C1r (plasma)	86,300	1.24
rMASP-2 (with Flag tag)	75,129	1.57

Figure. S2. SDS-PAGE analysis of HMGB1 digestion by C1s at 5% E/S ratio and of its HMGB1_KK variant between 0 and 25% E/S ratio in reducing conditions. * indicates bands corresponding to C1s.

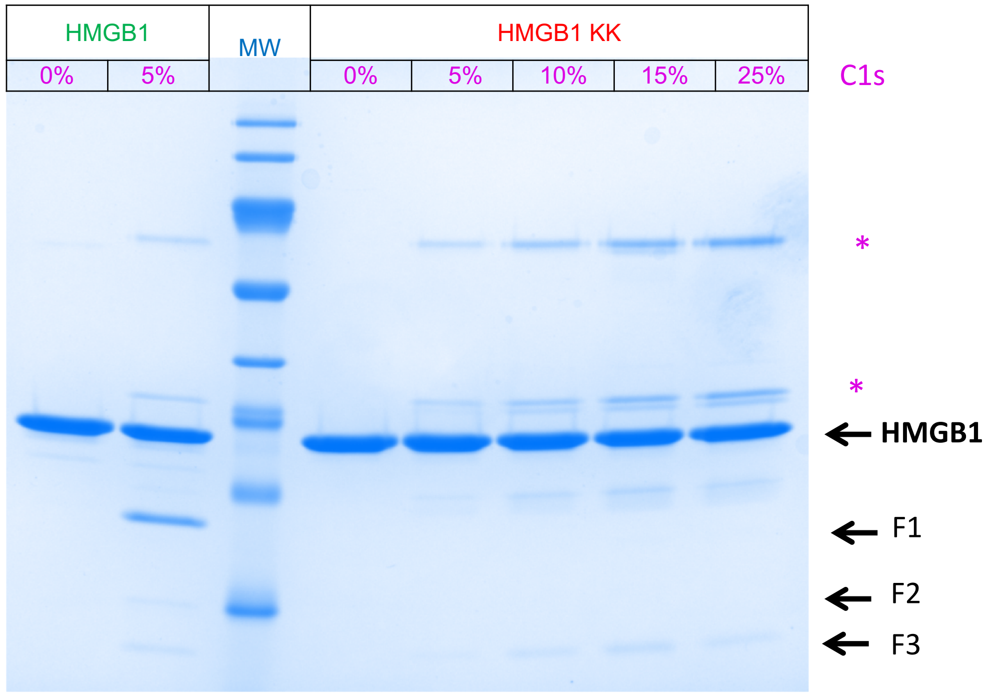

Figure S3. Experimental controls associated to Figure 8

A

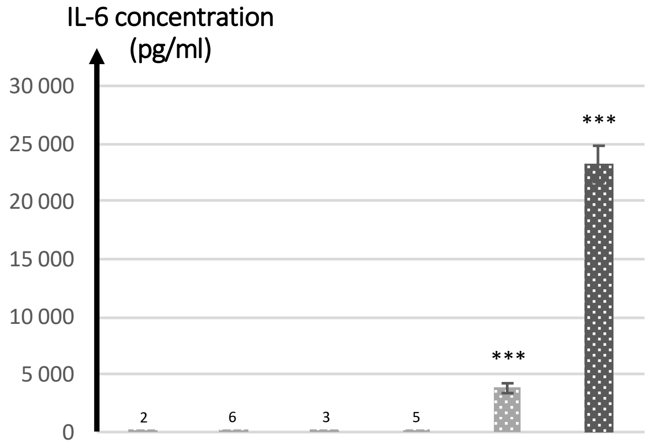

**HMGB1** (pmol/ml)	0	1.88	0	0	0	0
**rF3** (pmol/ml)	0	0	1.88	0	0	0
**A-box** (pmol/ml)	0	0	0	1.88	0	0
**LPS** (ng/ml)	0	0	0	0	1	2

This control experiment shows that HMGB1 or its fragments never triggered IL-6 secretion when used alone, even at the maximal concentration used.

B

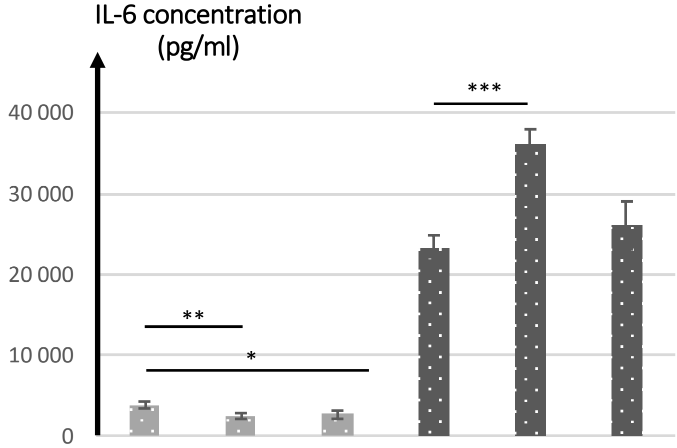

**HMGB1** (pmol/ml)	0	0.94	1.88	0	0.94	1.88
**LPS** (ng/ml)	1	1	1	2	2	2

This control experiment shows that HMGB1 enhances the IL-6 secretion when preincubated with LPS, as expected from previous reports.
* $p < 0.05$; ** $p < 0.01$, *** $p < 0.001$

Figure S4 (associated to Figure 8). Repeated observations supporting the significant functional difference between rF3 and the A-box impact on IL-6 secretion with LPS at low doses

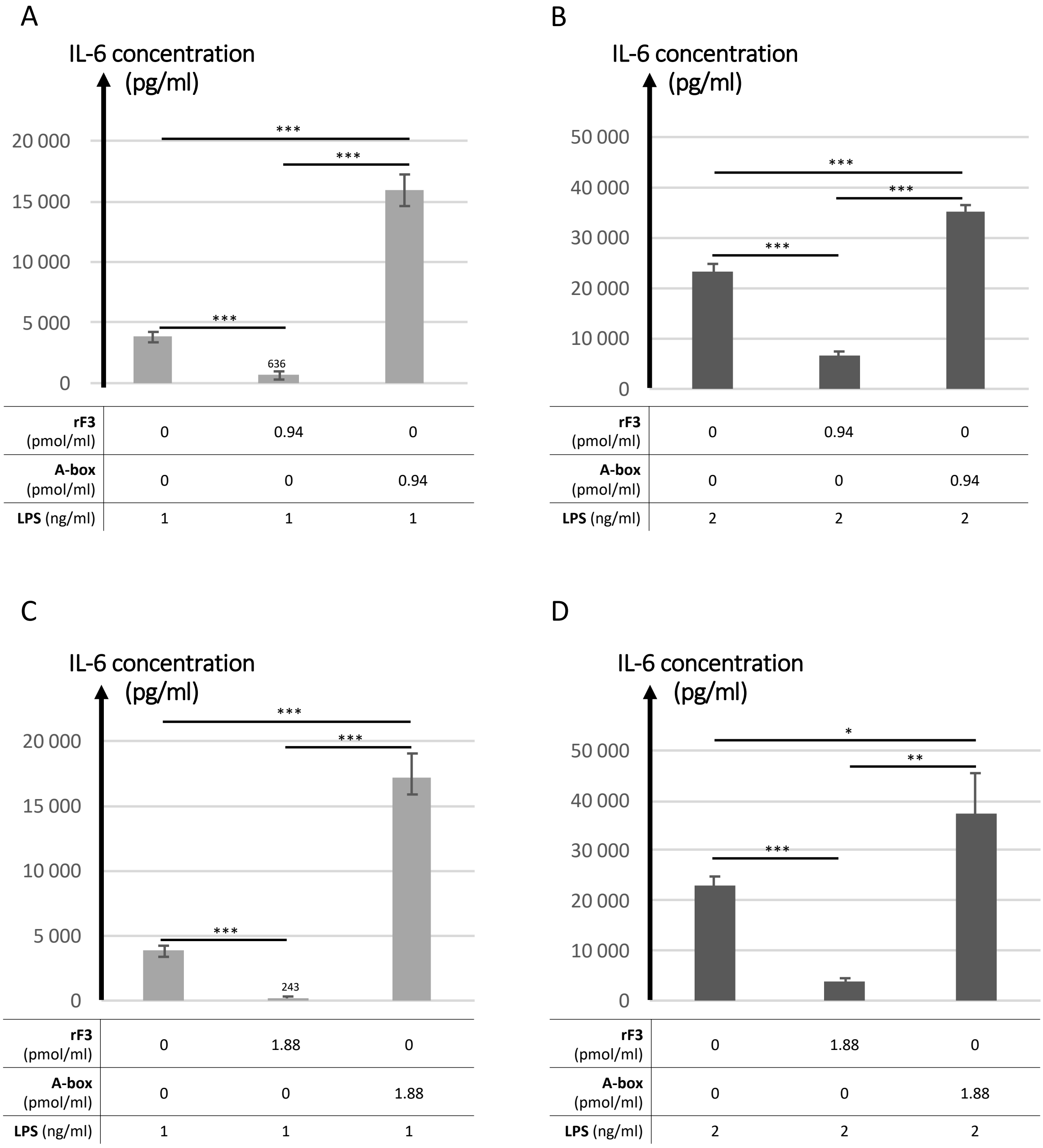

Results are presented as mean +/- standard deviations (n=4). The experiment presented here correspond to two independent replicates.
* $p < 0.05$; ** $p < 0.01$, *** $p < 0.001$

Figure S5 (associated to Figure 9). Complementary observations supporting the more effective digestion by C1s of the 3S variant, as compared to HMGB1 WT, with more F3 released.

The same experiment as the one reported in Figure 9 has been performed using incubation times increasing from 1 to 7 h. The tricine gels were scanned and the band intensity corresponding to HMGB, F1 and F3 is reported here, as a percentage of protein content.

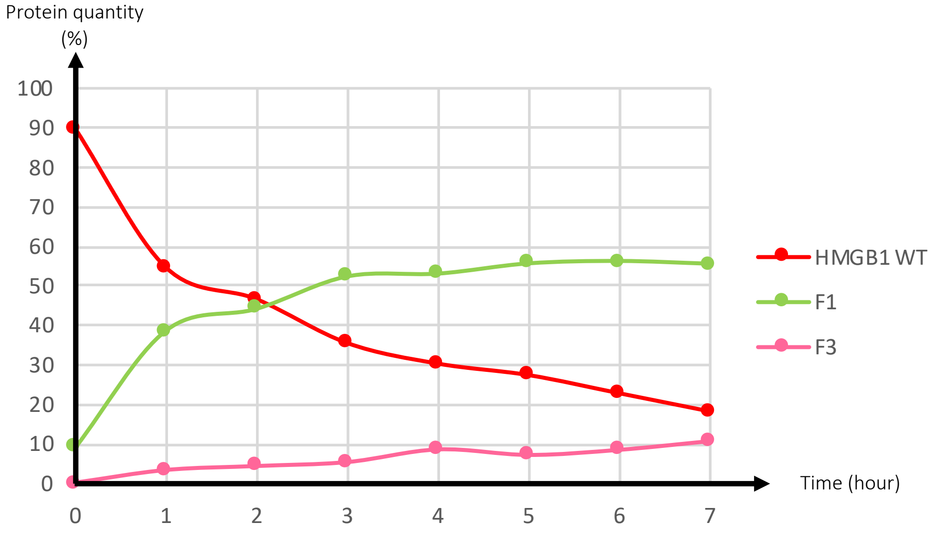

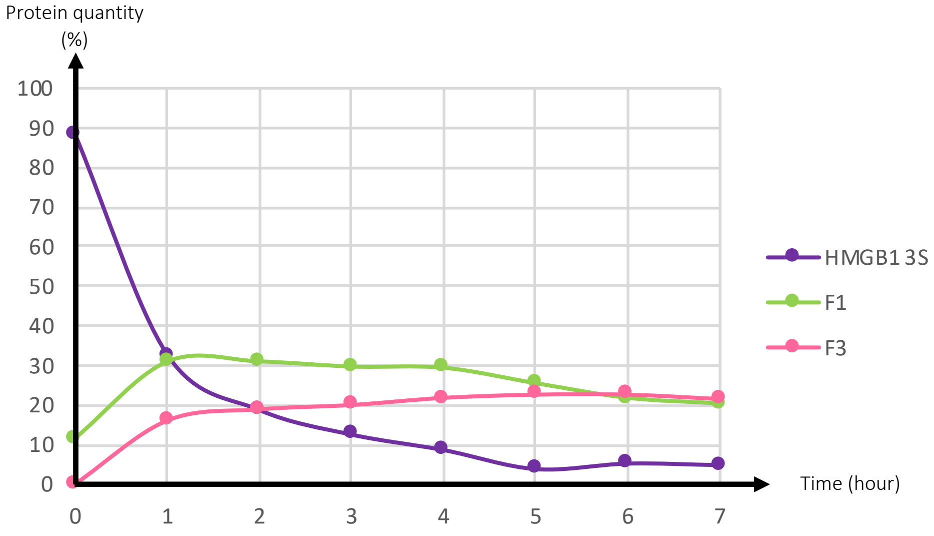

Supplement: Supplementary file 1 [file Presentation_1.pdf]
